# Insights into chlamydial infection at the sub-cellular level using label-free Raman spectroscopy in comparison to electron microscopy

**DOI:** 10.1016/j.jbc.2025.110940

**Published:** 2025-11-13

**Authors:** Nancy Unger, Elisabeth M. Liebler-Tenorio, Rustam R. Guliev, Simone Eiserloh, Sandor Nietzsche, Frauke Nowak, Sara Zuchantke, Christian Berens, Christiane Schnee, Ute Neugebauer

**Affiliations:** 1 Center for Sepsis Control and Care, Jena University Hospital, Jena, Germany; 2 Leibniz Institute of Photonic Technology, Member of Leibniz Health Technologies, Member of the Leibniz Centre for Photonics in Infection Research (LPI), Jena, Germany; 3 Friedrich-Loeffler-Institut – Federal Research Institute for Animal Health (FLI), Institute of Molecular Pathogenesis, Jena, Germany; 4 ThIMEDOP – CeTraMed, Jena University Hospital, Jena, Germany; 5 Center for Electron Microscopy, Jena University Hospital, Jena, Germany; 6 Friedrich Schiller University Jena, Institute of Physical Chemistry and Abbe Center of Photonics, Jena, Germany

**Keywords:** Raman spectroscopy, *Chlamydia abortus*, intracellular pathogen, non-invasive imaging, multivariate data analysis, transmission electron microscopy, N-FINDR analysis, elementary body, reticulate body

## Abstract

Intracellular infections are difficult to study as the host cell protects the pathogen from direct observation from the outside. Transmission electron microscopy (TEM) is the most commonly used method for subcellular analysis. However, sample preparation is based on fixation which prevents continuous observation. Here, we focus on the obligate intracellular bacterium *Chlamydia abortus*. It causes infections primarily in small ruminant livestock, and can also be transmitted to humans, where it can cause disease. Diagnosis is difficult, requiring PCR or cell culture. At the moment, non-invasive methods for the direct study of intracellular infections are rare and not yet established in routine analysis. In this study, we present 3D confocal Raman imaging as a non-invasive tool to investigate and characterize the infection directly inside intact host cells without the need for any purification step and compare the results to established conventional TEM. A 2D cell culture infection model with Buffalo Green Monkey kidney cells was employed and infection with *Chlamydiaabortus* S26/3 was characterized at different time points post infection. Using multivariate statistical data analysis, high-quality false color image stacks were generated from the Raman data. Two *Chlamydia* morphoforms, elementary body and reticulate body, could be distinguished based on their Raman spectral features: reticulate bodies are characterized by prominent lipid and nucleic acid signals while elementary bodies revealed higher carbohydrate and protein signals. This provides complementary information to TEM analysis where morphoforms are differentiated based on size and contrast. The complementary nature of both imaging methods is discussed in the manuscript.

Intracellular pathogens are particularly well-adapted to their hosts and have developed customized approaches for entering and exploiting the host cell to their advantage. As the bacteria are well sequestered in and adapted to their intracellular niche, they are often difficult to detect, to study and to treat (1). Thus, intracellular bacterial infections can pose a significant threat to human and animal health, often leading to severe diseases and economic losses in agriculture (2).

In this study, we focus on *Chlamydia abortus* as a widespread Gram-negative obligate intracellular pathogen that causes abortion and fetal death in mammals, especially in small ruminants such as sheep and goats, but also in larger animals (cattle, swine, horses, wild ruminants) and can result in significant economic losses in the livestock industry worldwide (3, 4). Rare zoonotic transmission to humans may lead to severe systemic infections with multi-organ failure, pneumonia and abortion (3, 5, 6). Recently, a new avian *C*. *abortus* subgroup has been increasingly described as an emerging causative agent in community acquired pneumonia cases in Asia (7, 8) and in the Netherlands (9, 10). Understanding the intricate dynamics of a *C*. *abortus* infection within the host cell is crucial for developing effective therapeutic strategies and preventive measures.

Conventional imaging techniques, such as immunofluorescence and electron microscopy, have provided valuable insight into intracellular localization and morphological changes during a *C*. *abortus* infection: Like all chlamydiae, *C*. *abortus* has a biphasic life cycle with two distinct morphoforms: a) the spore-like, metabolically less-active, but infectious elementary body (EB), and b) the replicative and metabolically active reticulate body (RB) (11). While these established imaging methods can provide valuable insight, they often require laborious sample preparation steps and lack the ability to provide native analysis of infected cells (12). Therefore, there is an urgent need for innovative imaging techniques that can overcome these limitations.

An emerging method to study intracellular pathogens directly inside intact host cells is Raman spectroscopy. It is based on the inelastic scattering of light. The frequency difference of incident and scattered photons provides detailed information about molecular vibrations, and, thereby, about overall chemical composition in a label-free, non-destructive and non-invasive manner (13, 14). This information serves as spectral fingerprint which can be used to differentiate not only between bacterial species (15), but also between metabolic states (16, 17). Chemical differences between the interior of the inclusion and the host cell cytoplasm have been revealed for endothelial cells infected with *Chlamydia trachomatis* (18). The potential of Raman spectroscopy to localize intracellular bacteria directly within intact host cells has been demonstrated previously for *Staphylococcus aureus* (19), *Coxiella burnetii* (20) and *Chlamydia psittaci* (21). In this study, we now advance and refine the method to a new pathogen not only with broader potential implication for zoonotic pathogen monitoring and for spectro-chemical differentiation of the two main morphoforms (EBs and RBs) of *C*. *abortus*, but also for elucidating the molecular and cellular basis of biological processes in a non-invasive manner. In this contribution, we directly compare findings by Raman spectroscopic imaging with established electron microscopy. Different parameters, such as sample preparation and information content of the two methods, are compared and discussed in the context of the intracellular life cycle of *C*. *abortus* in a 2D cell culture infection model using Buffalo Green Monkey (BGM) kidney cells, revealing the complementary nature of both methods. While Raman spectroscopy provides molecular-level insights (*e*.*g*., lipid/nucleic acid/carbohydrate/protein content) of the different morphoforms of *C*. *abortus*, TEM reveals ultrastructural details.

## Results

### Characteristic features of the two *Chlamydia abortus* morphoforms

We started our study with the characterization of the isolated morphoforms, *i*.*e*.*,* bacteria were removed from their native environment by destroying the host cells. For isolated bacteria, protocols for electron microscopy (negative contrast as well as scanning electron microscopy) and Raman spectroscopy are well-established. However, the destructive sample preparation might introduce artefacts and cannot reveal spatially resolved information within the host cell. Nevertheless, for Raman spectroscopy, analysis of isolated bacteria marks an important step, as the resulting spectra serve as reference data for the direct, non-destructive characterization of the *Chlamydia* morphoforms residing within intact eukaryotic host cells.

#### Electron microscopy

**Figure 1:**
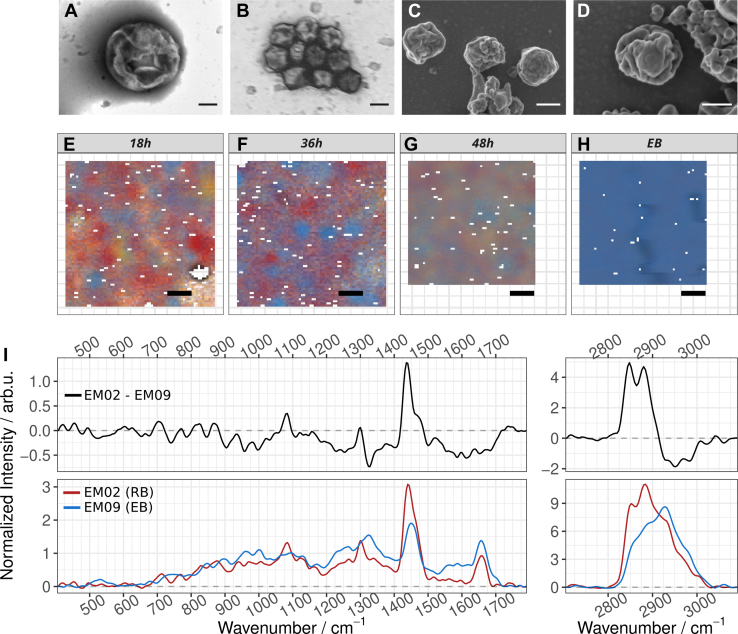
**Analysis of isolated *C*. *abortus* morphoforms**. *A* through *D*, electron microscopy: *A*, negative contrast TEM image of an RB, *B*, negative contrast TEM image of a group of EBs. *C*, scanning electron microscopy image of EBs using the same isolation protocol as for Raman characterization. The EB shows a fissured surface and a size of approximately 300 nm (magnification: 8,000×). *D*, higher magnification of an EB (magnification: 10,000×). The scale bars represeent 200 nm. *E* through *H*, false color Raman images of drop casted isolated morphoforms. *Blue*: EB, Red: RB, *yellow/green*: Background. White pixels had to be removed during preprocessing to remove spectra with cosmic spikes. The scale bar represents 1 μm, pixel size: 87 nm × 87 nm. (Further images, also in *gray scale* are available in Supplementary Fig. S1*B*). *E*, isolated *C*. *abortus* 18 h post infection. RBs (*red*) dominate the isolated bacteria. In addition, several EBs (seen as *blue clusters*) are visible as well. *F*, isolated *C*. *abortus* 36 h post infection. RBs (*red*) still dominate the isolated bacteria, but more EBs are present than at 18 h (panel E). *G*, isolated *C*. *abortus* 48 h post infection. Both, RBs (*red*) and EBs (*blue*) are present. *H*, isolated *C*. *abortus* with protocol optimized to isolate EBs. *I*, *bottom*: Endmember spectra assigned to RB (*red*) and EB (*blue*). *Top*: Computed difference spectrum (RB minus EB) between the chlamydial morphoforms, *Positive bands* highlight Raman features that are more prominent in RBs, negative bands those that are more prominent in EBs. EB, elementary body; RB, reticulate body.

Figure 1, *A* and *B* depict negative contrast transmission electron microscopy (TEM) images of isolated reticulate bodies (RBs) and elementary bodies (EBs), respectively. It is clearly visible, that the metabolically active RBs are much larger than the infectious EBs. RBs presented as spherical to ovoid structures with diameters between 500 nm and 1000 nm, while the smaller EBs were spherical with diameters between 200 nm and 300 nm. Both, RBs and EBs, had an irregular surface and were surrounded by a loosely folded, wavy Gram-negative cell wall. This corrugated surface was most likely caused by the aldehyde fixation step which resulted in shrinkage of the bacterial cytoplasm.

A sample of isolated EBs (using the same isolation procedure as for Raman spectroscopic characterization) was subjected to scanning electron microscopy. Figure 1, *C* and *D* depict the respective images with 8000× and 10,000× magnification, respectively. Individual spherical particles with a diameter of roughly 300 nm showed a fissured surface similar to that already visible in the negative contrast images (Fig. 1*B*). Size information obtained from our samples agrees with published values from other studies (11, 22). Also, fissured surfaces of the isolated bacteria have been shown in electron microscopy images from *C*. *trachomatis* (23).

#### Raman spectroscopic differentiation

Raman image scans of isolates of the two *C*. *abortus* morphoforms EBs and RBs were recorded with a small step size (∼87 nm/pixel) and mathematically unmixed using the N-FINDR algorithm. Spectra of all endmembers are displayed in Supplementary Figure S1*A*. Based on their spectral characteristics and spatial abundance in the false color Raman images (Fig. 1, *E*–*H*, and Supplementary Fig. S1*B*), two endmember spectra were assigned as RB (endmember EM02) and as EB (endmember EM09), respectively, and are displayed in Figure 1*I*. Both spectra show typical characteristics of bacteria, in particular Raman bands around 1655 cm^-1^ as well as around 1440 cm^-1^, originating from the C=O stretching vibration of the amide I band (1655 cm^-1^), which is characteristic for proteins, as well as from C-H deformation vibrational modes (1440 cm^-1^) of proteins, lipids and carbohydrates. Both spectra also present characteristic features which are unique to the particular morphoform (see also Table 1 for detailed assignment). For RBs, in particular, the sharp Raman bands around 1300 cm^-1^ and 1085 cm^-1^ stand out. They can be assigned to the CH_2_ wagging mode of lipids (1300 cm^-1^) as well as the symmetric PO_2_^-^ stretching vibrations of the RNA backbone and of phospholipids (1085 cm^-1^). Further spectral features in the Raman spectra of RBs also point to a high abundance of nucleic acids in this metabolically active morphoform: the phosphate backbone vibration of RNA shows a band around 420 cm^-1^, the Raman band around 450 cm^-1^ can be assigned to DNA and RNA backbone vibrations, and at 710 cm^-1^, 768 cm^-1^ and 825 cm^-1^, the ring breathing modes of the nucleic acid bases are visible. A high lipid content in RBs is not only suggested by the Raman band at 1300 cm^-1^, but also by the Raman bands around 1740 cm^-1^ originating from the ester C=O stretching vibration as well as the prominent CH stretching vibrations at 2850 cm^-1^ and 2885 cm^-1^.

**Table 1:** Assignment table of spectral features (first column) predominantly found in the metabolically active RBs (second column) and those predominantly found in the robust EBs (third column)

Raman band (cm^-1^)	Assignment of dominant Raman bands present in RBs	Assignment of dominant Raman bands present in EBs
420	Phosphate backbone RNA	
450	DNA/RNA backbone	
530		S–S stretch — cysteine-rich outer membrane protein crosslinks (rigid envelope)
675		C–S stretching (cysteine/methionine side chains in OMPs)
710	Adenine ring breathing (ATP, RNA)	
768	Uracil/cytosine ring breathing (RNA marker)	
825	RNA pyrimidine ring breathing	
845		C–O–C glycosidic stretch (outer-layer carbohydrates, tyrosine side chains)
875	C–O–P stretch (phosphate esters, RNA; glycogen)	
890–900		Carbohydrate vibrations, glycogen
960		Phosphate esters (C–O–P vibrations), *e*.*g*.*,* in phosphorylated sugars or condensed phosphates
1000	Phenylalanine (active protein biosynthesis)	Phenylalanine (structural proteins in OMPs)
1085	Symmetric PO_2_^-^ stretch (RNA backbone and phospholipids)	
1230–1340	Amide III (proteins)	Amide III (proteins)
1300	CH_2_ wagging (lipids)	
1325		CH_2_/CH_3_ wagging, structural backbone, glycogen
1440	CH_2_/CH_3_ deformation — protein dominated	CH_2_ deformation — lipid + protein backbone
1650	Amide I (α-helix, protein synthesis)	Amide I
1680–1690		Amide I, β-sheet/crosslinked proteins (rigid OMPs)
1740	Ester C=O stretching in lipids	
2850	CH_2_ symmetric stretching (lipids)	
2885	CH_2_/CH_3_ stretching (saturated and ordered/closely packed lipid acyl chains)	
2930		CH_3_ symmetric/asymmetric stretch (proteins)

EB, elementary body; RB, reticulate body.

In the Raman spectra of EBs, the prominent features of lipids and nucleic acids are missing. Instead, the Raman spectrum is dominated by spectral characteristics of proteins and carbohydrates. The amide I (1650 cm^-1^) and amide III (1230–1340 cm^-1^) bands, but also the phenylalanine band at approximately 1000 cm^-1^ as well as the CH_3_ stretching vibration (2930 cm^-1^) are very strong. The Raman band at roughly 530 cm^-1^ indicates the presence of S-S stretching vibration from disulfide bonds. The band around 675 cm^-1^ can be assigned to C-S stretching vibrations, *e*.*g*.*,* from sulfer-rich amino acids, such as cysteine and methionine.

The computed difference spectrum (RB minus EB, Fig. 1*I*) visualized the differences between the two *Chlamydia* morphoforms in another representation. Positive bands indicate features more prominent in RBs while negative bands highlight spectral features that are more prominent in EBs. RB spectra are dominated by Raman signatures from lipids and nucleic acids, which is in good agreement with their metabolically active state involving transcription and nucleotide biosynthesis (11, 24), as well as a fatty acid and phospholipid metabolism which is known to be supported by an active uptake of eukaryotic lipids such as sphingolipids, cholesterol and glycerophospholipids for growth and development of the intracellular *Chlamydia* (25). The spectral features of the metabolically inactive EBs point to a prominent presence of proteins, which also show signs of cross-linking, as well as the presence of more carbohydrates. The amide I band, which is primarily associated with the C=O stretching vibration of the protein backbone, has a different shape in RBs and EBs indicating a different protein composition. Those findings are in agreement with the fact that EBs contain glycogen and have a more robust and cross-linked peptidoglycan layer compared to the less rigid layer in RBs (22). EBs hardly show any nucleic acid vibrations. This can be explained by the condensed genome in the metabolically inactive morphoform. Reduced or absent nucleic acid Raman signals upon dense DNA packing have also been observed in earlier studies with other bacteria, in which nucleic acid marker bands contributed to the differentiation between actively growing bacteria in the logarithmic growth phase and those in the stationary phase (26).

In the false color images of the Raman maps of dried isolated bacteria (Fig. 1, *E*–*G*), individual RBs can be identified as red roundish bodies with an approximate diameter of 500 nm to 1 μm. This agrees with the findings from electron microscopy (Fig. 1*A*). It is also visible that the isolated fractions at the different isolation time points (Fig. 1*E*: 18 h, Fig. 1*F*: 36h, Fig. 1*G*: 48 h) contain not only RBs, but that some contributions from EBs are also present. Sometimes, those EB-associated features also have a diameter of 1 μm, which can be explained with clusters of several EBs, as is also depicted in the EM image in Figure 1*B*.

### Life cycle analysis

#### Transmission electron microscopy (TEM)

**Figure 2:**
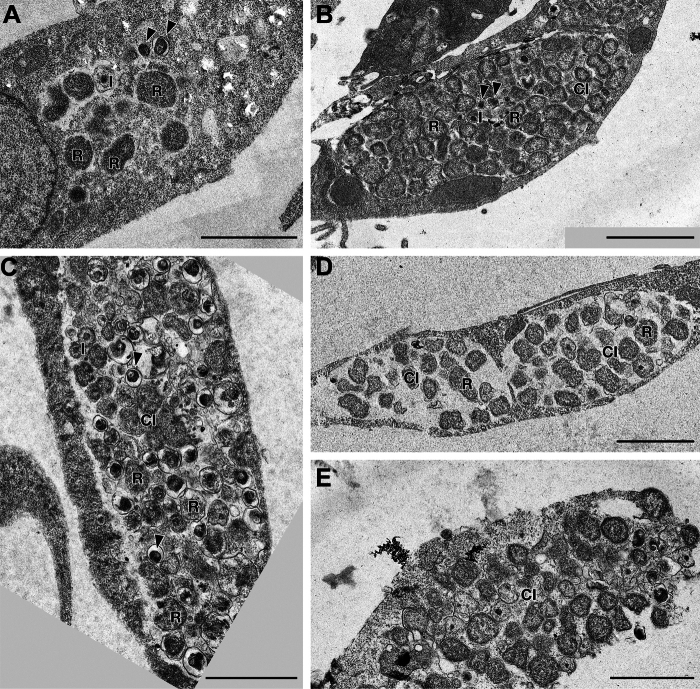
**Examples of chlamydial inclusions**. *A*, small inclusions with RBs (R, exempl.), a few EBs (*arrowheads*, exempl.), and an intermediate body (I) at 18 h p.i. *B*, single, large inclusion (CI) filling most of the cytoplasm and causing mild distension of the cell at 36 h p.i. RBs (R, exempl.) predominate, EBs (*arrowhead*, exempl.) and IB (I, exempl.) are less frequent. All morphoforms are closely packed in the CI. C, A single large CI, slightly smaller than at 36h, filled with a mixture of RBs (R, exempl.), EBs (*arrowheads*, exampl.), and IBs (I, exempl.) at 48 h p.i. *D* and *E*, highly variable chlamydial inclusions (CI) at 54 h p.i. Some CIs are loosely filled with RBs (R, exempl.) comparable to CIs at 18 h p.i. *D*, others are disintegrated *E*, The scale bars represent = 2 μm. More images are shown in Supplementary Figure S2. EB, elementary body; RB, reticulate body; CI, chlamydial inclusion.

Most cells had inclusions containing *Chlamydia* at all time points of the life cycle examined (18 h, 36 h, 48 h, 54 h). Both, number and shape of the chlamydial inclusions (CI), and the absolute and relative frequency of morphoforms within the inclusions varied. The high resolution of TEM allowed discriminating another morphoform, intermediate bodies (IB), in addition to EBs and RBs (Fig. 2, Supplementary Table S1). EBs were round, electron-dense and 200 to 300 nm in diameter. Sometimes, a clear periplasmic space separated plasma membrane and outer membrane. IBs were also round, slightly larger (400–500 nm in diameter) and moderately electron-dense with a small electron-dense core. RBs were larger than EBs and IBs (>800 nm), round to oval to pleomorphic in shape and had an even, moderately electron-dense, finely granular cytoplasm. The number of inclusions per cell, and the absolute and relative frequencies of EBs, IBs and RBs per cell and per inclusion were counted and calculated (Fig. 4*A*, Supplementary Table S2).

At 18 h, CIs were small, sometimes containing only a single chlamydial cell, which caused only minimal distension of the host cells (Fig. 2*A*, Supplementary Fig. S2*A*). Frequently, several pleomorphic CIs, not clearly delineated from the vacuolated host cell cytoplasm, were present in one cell. They were interpreted as CIs in the process of fusing. In addition, a few well-defined CIs, loosely filled with *Chlamydia* and bordered by a distinct inclusion membrane, were seen and interpreted as the result of the fusion process. RBs were most frequently, often exclusively, present in CI, EBs only rarely, and IBs even more seldom (Fig. 2*A*, Supplementary Fig. S2*A*).

At 36 h, CIs had markedly increased in size (Fig. 2*B*, Supplementary Fig. S2*B*). In most cells, one large inclusion filled most of the cell’s volume and indented the nucleus displacing it to the cell’s edge. Cells were distended by the CI, but many retained an elongated shape and rounded cells were rare (Fig. 2*B*, Supplementary Fig. S2*B*). Occasionally, more than one CI was present. Most CIs were crowded by *Chlamydia* with no or minimal space between individual bacteria. RBs were still most frequent, but in some CIs, a single or a small group of EBs and/or IBs were seen besides the RBs (Fig. 2*B*, Supplementary Fig. S2*B*). The median of EB relative frequency was lowest at 36 h, compared to 18 h, and also all other observation times (Fig. 4*A*).

At 48 h, single large CIs, which were slightly smaller than at 36 h, were most frequently seen (Fig. 2*C*, Supplementary Fig. S2*C*). All CIs contained a mixture of RBs, EBs and IBs, but the frequencies of the individual morphoforms varied (Figs. 2*C* and 4*A*). Overall, the absolute and relative numbers of RBs were higher than those of EBs and IBs, but the percentage of RBs at 48 h was lower than at 18 h and 36 h (Fig. 4*A*). Bacteria were more loosely arranged in CIs when more EBs and IBs were present (Fig. 2*C*), while they were more densely packed in CIs predominantly containing RBs. Occasionally, loss of the CI membrane and disintegration of the host cell were observed.

At 54 h, CIs were generally smaller than at 36 h and 48 h p.i. and highly variable in size as well as in the composition of the enclosed morphoforms (Fig. 2, *D* and *E*, Supplementary Figs. S2*B*, S4*A*). Multiple small CIs in vacuolated host cell cytoplasm and small CIs loosely filled with RBs, as observed at 18 h (Fig. 2*D*), large CIs densely filled with RBs like at 36 h and large CIs containing a mixture of morphoforms like at 48 h were all present. There was a high number of disintegrated cells with disintegrated CIs in which morphoforms could not always be identified unequivocally (Fig. 2*E*), indicating release of *Chlamydia* and initiation of new life cycles.

#### Three-dimensional visualization and reconstruction of the *Chlamydia abortus* life cycle within intact host cells using Raman spectroscopic imaging

**Figure 3:**
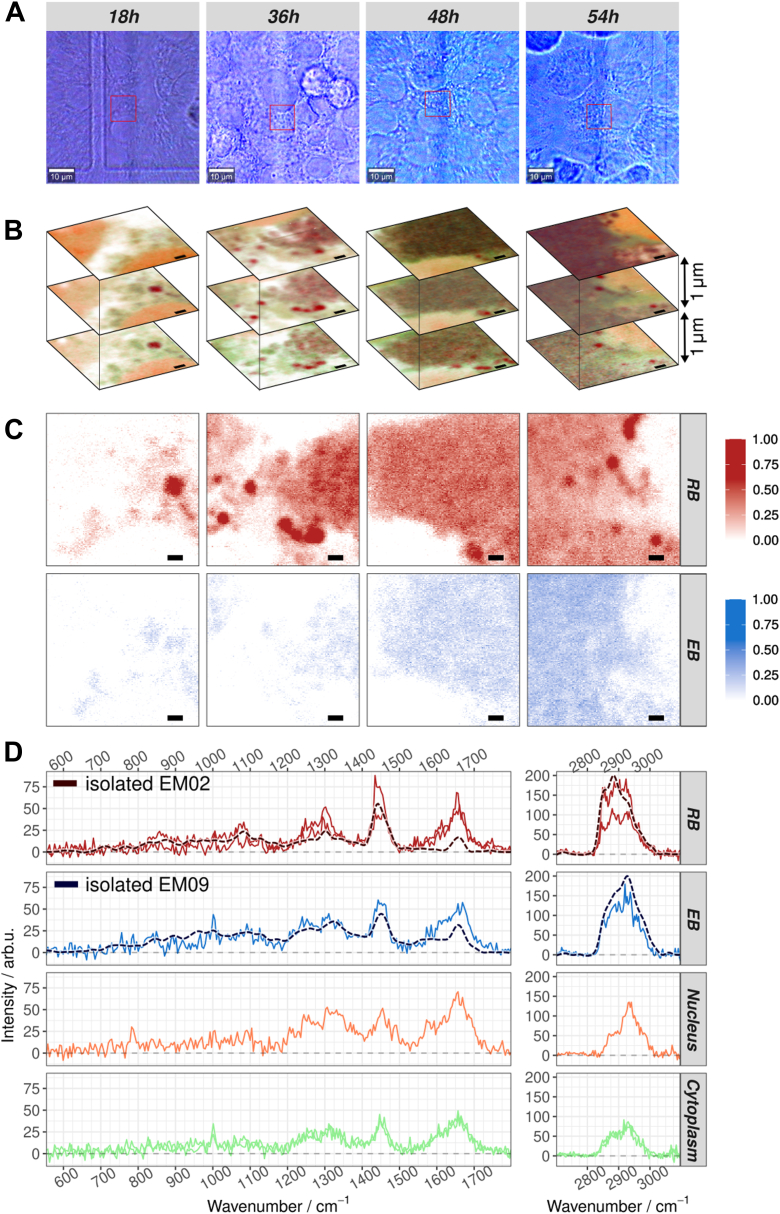
**Analysis of 3D Raman image stacks of infected BGM cells at time points 18, 36, 48 and 54 h post infection (p.i.)**. *A*, bright field overview images of BGM cells infected with *C*. *abortus* at different hours p.i. The *red square* highlights the region (10 μm × 10 μm) that was further analyzed by Raman spectroscopic imaging. This scale bar represent 10 μm. *B*, false color Raman images of the three z-planes measured in the region highlighted in panel *A*. Blurring filter and 3D perspective were applied for better visualization. The color code is the same throughout the *panelsB*–*D*: *white* - PBS/water, *orange* - DNA/nucleus, *dark red* - RB of *C*. *abortus*, *mid blue* - EB of *C*. *abortus*, *light green* - cytoplasm of the host cell. The false color image is a superposition of the relative abundance of each endmember in each pixel. The scale bars represent 1 μm. The distance between z-layers is 1 μm. *C*, *gray-scale* maps showing abundance distributions of *C*. *abortus* endmembers in the middle layer shown in *panel B*. Color gradients were corrected to make lower values more visible. *D*, endmember spectra corresponding to the abundances shown in *panelsB* and *C*. For *C*. *abortus*, the spectra are overlaid with the corresponding endmember spectrum of isolated bacteria from Figure 1*I* for comparison. The spectra of isolated *C*. *abortus* are upscaled to simplify visual comparison. Original values are provided in Figure 1*I*. Supplementary Figures S3, and S4 provide the same information for all eight Raman images without additional effects. BGM, buffalo green monkey; EB, elementary body; RB, reticulate body.

Raman micro-spectroscopic analysis of infected host cells was performed at time points post infection equivalent to the electron microscopy data, *i*.*e*.*,* at 18 h, 36 h, 48 h, and 54 h p.i. Representative bright field images of regions of interest are shown in Figure 3*A*. Raman images were recorded with a diffraction-limited spatial resolution of roughly 69 nm in xy in three different z-planes with a step size of 1 μm in z-direction. Using the N-FINDR algorithm for spectral unmixing, false color Raman images was generated. They reveal the two bacterial morphoforms, EB and RB, as well as contributions from the host cells, such as cytoplasm or nucleic acids in the host cell nucleus, in each image plane and in the 3-layer z-stack (Fig. 3*B*).

The spatial resolution of the Raman imaging approach allowed localization and visualization of the bacteria directly within the intact host cells in 3D image stacks without the need for any labelling or slicing of the cell. Figure 3*C* and Supplementary Figure S4 highlight the spatial distribution of RBs and EBs, respectively, for the false color image stacks shown in Figure 3*B*.

Assignment of the individual endmember spectra to the biological components inside the intact host cell is based on their spectral characteristics (Fig. 3*D*). The nucleic acid/DNA endmember shows characteristic nucleic acid base vibrations, *e*.*g*.*,* cytosine ring breathing mode at 785 cm^−1^, adenine/guanine vibrations around 1485 cm^−1^ as well as adenine/guanine ring stretching vibrations around 1575 cm^−1^. In the false color Raman images, it is found in regions of the host cell nucleus as well as inside the *C*. *abortus* inclusion. EB and RB were each assigned an individual endmember. As shown in the overlay in Figure 3*D*, they reflect very well the spectral characteristics of the isolated bacterial morphoforms presented earlier (Fig. 1*I*). In particular, RB spectra show the sharp and very prominent Raman bands around 1300 cm^-1^, 2850 cm^-1^ and 2885 cm^-1^ as well as the small ester vibration around 1740 cm^-1^, which were all characteristic for the high lipid content in RBs. For EBs, these features are missing, carbohydrate/glycogen and protein bands dominate the spectrum (Table 1).

**Figure 4:**
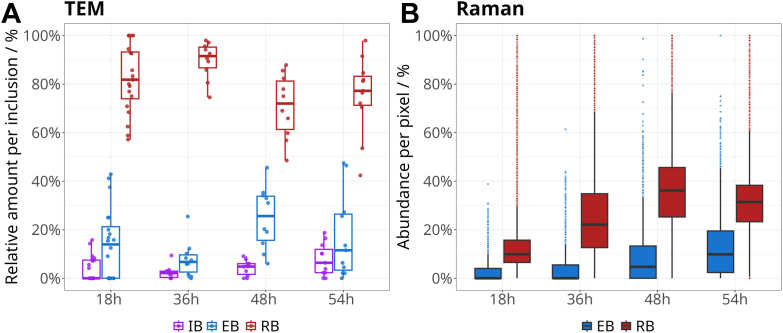
**Quantitative analysis of intracellular chlamydia.***A*: from TEM micrographs. Relative numbers of morphoforms per chlamydial inclusion (CI). RBs (*red*), EBs (*blue*), IBs (*purple*). RBs predominate at all time points, EBs and IBs are present with increased frequency at 48 h and 54 h (data are displayed in Supplementary Table S2). *B*: from Raman spectroscopy. Distribution of RB (*red*) and EB (*blue*) endmember abundances in Raman images (*i*.*e*.*,* each point is a pixel) combining all eight Raman 3D image scans. Abundance values are scaled from 0 to 1 to 0 to 100 to be represented in percentages. The images were masked so that only pixels with less than 5% of total RB and EB contribution were excluded, *i*.*e*.*,* excluding irrelevant PBS/water and nucleus regions. Applied masks are shown in Supplementary Figure S4. Boxplots in both panels *A* and *B* illustrate data variability, with the box spanning the interquartile range (IQR) from Q1 (25%) to Q3 (75%), the line inside marking the median (Q2), and whiskers extending from Q1 − 1.5×IQR to Q3 + 1.5×IQR. *Dots indicate*: *A*, all individual data points and *B*, only pixels outside the whisker range; other pixels are not shown, as over 10^4^ pixels contributed to each time point. An alternative histogram representation of the data in *panelB* is provided in Supplementary Figure S6. EB, elementary body; CI, chlamydial inclusion; RB, reticulate body.

The false color images have been analyzed quantitatively for all Raman image stacks to compare them with the insights gained from the analysis of the TEM data. Figure 4 shows a similar trend for the abundance of EBs and RBs at the different time-points post-infection in the analyzed images. It has to be noted that due to technical reasons, the analysis was different for the TEM and Raman data. While for TEM images, the number of the individual *C*. *abortus* morphoforms could be directly counted per inclusion in the high-resolution images, this was not feasible for the Raman data due to the small size of the bacteria (at and below the diffraction limit). Nevertheless, with the N-FINDR unmixing algorithm, it is possible to calculate the relative abundance of each characteristic endmember spectrum, here, in particular of EB and RB, in all pixels of the chlamydial inclusion. RBs predominate at all time points (Fig. 4*B*), which is also observed in the TEM results (Fig. 4*A*). EBs (and in the TEM images also IBs) are present with increased frequency at 48 h and 54 h (Fig. 4).

## Discussion

In this study, we applied two complementary imaging techniques, namely, conventional TEM and label-free Raman imaging, to visualize and characterize intracellular infections of *C*. *abortus* in a cell culture infection model at different time points post infection. With its biphasic developmental cycle and the two major morphoforms, EBs and RBs, differing both in size and chemical composition, *C*. *abortus* is a perfect model system for a comparison of the strengths and shortcomings of the two different imaging methods (Table 2). The smaller EBs with a spherical size and a diameter of roughly 200 to 300 nm are close to or below the spatial resolution limit of the diffraction-limited optical imaging used in Raman imaging. With a Raman excitation wavelength of 532 nm and an objective with a numerical aperture of NA 1, a diffraction-limited spatial resolution in the xy-plane of > 266 nm can be achieved with perfect alignment. The larger RBs with a diameter of approximately 500 to 1000 nm are well above the diffraction-limited spatial resolution of Raman imaging and, thus, should be clearly resolved. This was also achieved for the dried isolated morphoforms in which individual RBs could be visualized on the image scan. The smaller EBs were only visible as clusters. Due to the much smaller De Broglie wavelength of the electrons, spatial resolutions of < 0.1 nm can be achieved with electron microscopy, which even enables the visualization of the ultrastructure of the morphoforms. Clear differences in the size and shape of EBs and RBs were used to characterize and differentiate them. In electron microscopy images, a third morphoform, the intermediate bodies (IBs), were additionally detected. While IBs are distinct in the EM images, this low abundance transitional stage between RBs and EBs, where RBs begin condensing into the infectious EBs, is difficult to isolate physically.

**Table 2:** Comparison of conventional transmission electron microscopy (TEM) and Raman spectroscopy for the analysis of intracellular bacteria

Parameter	Conventional TEM^a^	Raman spectroscopic imaging
Condition of sample	Chemically fixed, embedded in resin (60 nm sections)	In this work: fixed intact cells, but live cell imaging is also possible
Sample preparation	Destructive; Duration: days to weeks	Native, intact sample, minimal preparation, ideally on low-background slide (CaF_2_, quartz)
Speed of data acquisition	<1 s for micrographs of selected areas	∼ 1s/pixel (2 h per image: 6 h per stack) for current point imaging
Spatial resolution	< 0.1 nm in one plane	Diffraction-limited: d_xy_ > 266 nm, d_z_∼500 nm (for excitation with 532 nm)
Data analysis	Visual identification, image analysis	Multivariate statistical data analysis
Information content	Morphology of host cells and chlamydial inclusions; number, size and morphology of morphoforms	Label-free insights into biochemical composition, size and shape (within diffraction limit). False color images Differentiation between different morphoforms possible, including quantitative analysis

Both methods are able to generate insightful images that yield information on the composition of the chlamydial inclusions. (A more extensive form of the table is available as Supplementary Table S3).

TEM, transmission electron microscopy.

a More advanced methods, such as HRTEM, STEM, Cryo Substitution TEM, TEM tomography, and Atomic Force Microscopy are not considered.

With a diffraction-limited spatial resolution of roughly 300 nm, Raman spectroscopy cannot rely on size and shape information to differentiate the bacterial morphoforms. Imaging contrast is obtained from different inelastically scattered light on different molecules with different vibrational modes. Thus, with Raman spectroscopy, the imaging contrast yields insights into chemical composition. As an intrinsic molecular property of the molecules (molecular vibrations) is probed, no labelling or contrasting is needed, making sample preparation straight-forward, fast and easy. We showed that characteristic chemical differences, such as a higher content of lipids and nucleic acids in the metabolically active RBs and different protein expression profiles in EBs and RBs could be used to reliably differentiate these. This was possible, not only when the bacteria were mechanically isolated from their host cells, but also when they were investigated directly inside intact host cells. The quantitative analysis of the Raman images was carried out pixel by pixel with unmixing the spectral contributions of the reference spectra (RBs and EBs), whereas with TEM, the bacteria in the images could be counted individually. IBs were not specifically detected in the Raman false color images. In the liquid-filled inclusions inside the intact host cell, it was not possible to spatially resolve the small bacteria. As also not purified isolates were available, it was not possible in this study to assign characteristic chemical features to this transition that unambiguously separates IBs from the other two morphoforms. In an earlier Raman study on *Protochlamydia amoebophila*, IBs could also not be differentiated and it was concluded that the chemical composition of IBs is either assigned to RBs or EBs by Raman (16). In neither study, isolated IBs were available for pure spectrum recording, likely due to difficulties in preparing this morphoform in a pure culture.

Both imaging methods, conventional TEM and Raman imaging, are not only capable of providing high quality images, but also to extract quantitative information on the infection status (Fig. 4). It has to be noted, that the imaged samples differed for both methods due to the specific sample preparation requirements. Thus, a direct comparison of the quantitative values can only be done by comparing averages of different CIs. Nevertheless, the overall trend is quite comparable for both imaging methods.

While sample preparation is quite simple, short and straightforward in Raman imaging (taking only a few minutes), image acquisition can be quite time consuming with the currently available point mapping spectrometers, especially if many image points have to be acquired for high resolution imaging of a significant fraction of the chlamydial inclusion or even the whole cell. Imaging times of several hours for a z-stack can easily become the bottle neck for the acquisition of high numbers of images per time point. True imaging Raman devices that can capture the Raman information in the xy plane in one shot would speed up the characterization significantly and provide a huge benefit. These devices are currently under development, but are not yet readily available to study biological samples. In a few laboratories, line-scanning devices are used to speed up data collection.

In contrast, while for TEM imaging, sample preparation can be quite time consuming (up to 2 weeks), imaging is almost in real-time, making it relatively easy to acquire several image sections from the same sample. This is a true advantage, if heterogeneous events such as CIs at 54 h p.i. have to be analyzed.

An important advantage of Raman imaging over conventional TEM imaging is that Raman spectroscopy is label-free, non-destructive and suitable for 3D live cell imaging. Thus, perspectively, a live cell experiment can be designed in which the very same cell with its unique chlamydial inclusion can be followed in time by Raman spectroscopy. Due to technical restrictions, this has not been exploited in the current study, but extended live-cell imaging results have been published for other experimental setups (27, 28).

Taken the specific characteristics of the imaging methods into account, we could demonstrate that Raman spectroscopy can provide valuable information on the *C*. *abortus* intracellular state in a non-destructive manner resulting in diffraction-limited 3D false color images, insights into chemical composition and quantitative information. This information complements results from analysis of TEM images where morphological characteristics are revealed at high (sub-single bacteria) resolution. Due to the complementary nature of the methods, imaging the very same sample with both methods would be of utmost interest. As the sample has to be measured in vacuum for electron microscopy, live cell imaging is not possible in a joint “bi-modal imaging” experiment. Thus, for a combination of both methods, two approaches are tentatively feasible: in the first approach, Raman characterization would be performed first using live cell imaging. After data acquisition, the sample would be fixed with glutaraldehyde, dehydrated and covered with resin. After performing short-time polymerization, the sample can be detached from the glass slide and established electron microscopy steps can be performed (29). As this is very time-consuming, especially when the very same position where the Raman data were acquired, has to be found, the second approach aims for technological development to combine Raman imaging and scanning electron (RISE) microscopy within one device (30). In this option, the advantage of live cell imaging with Raman spectroscopy has to be sacrificed, but it is relatively easy to localize the very same sample position. Due to the requirements for sample preparation, this approach has so far mainly been used for the advanced characterization of different surfaces, such as graphene, MoS_2_ or mineral surfaces from geosciences (31) with only very few applications from life sciences (32).

Our study primarily focuses on pathogen-specific morphological and chemical changes during bacterial growth, but pays limited attention to potential host cell interference. This would be an interesting aspect for future research. Here, we could follow the replication of *C*. *abortus* inside the host cell from small inclusions filled with single bacteria at 18 h p.i., to larger inclusions full of RBs and with the major transition of RBs to EBs starting at approximately 48 h p.i. These observations are in agreement with literature data, in which the first EBs were reported after 48 h p.i. in a TEM study (33). At 54 h p.i., a highly heterogeneous infection status is found with the co-existence of RBs and EBs within the same inclusion. While in one inclusion, EBs already start to become the predominant morphoform, in another inclusion, RBs were still the dominating morphoform. This points to a non-synchronized infection where inclusions may be of differing ages. Future research could try to put these bacteria-associated changes into relation of host factors, such as mitochondria translocation.

## Conclusions

In this contribution, we showed that Raman spectroscopic imaging and conventional transmission electron microscopy can be used to reveal matching and in part complementary information on the infection process of *C*. *abortus*. Similar Raman-based analysis has been done earlier for other intracellular bacteria (*e*.*g*.*, S*. *aureus* (19), *C*. *burnetii* (20) and *C*. *psittaci* (21)) some of which have different intracellular life cycles and biochemical characteristics. This suggests that label-free Raman imaging could evolve to a powerful, robust and reliable method to study obligate intracellular bacteria which are often difficult to assess in their native state within the host cell. Demonstrating reliable visualization and characterization of various intracellular pathogens with different intracellular life cycles, highlights the adaptability of the Raman method to fulfill needs from different scientists to undertake their biochemical and cell biological inquiries in different contexts. With the spectroscopy-based insights on *C*. *abortus*, the study offers now new applications of the Raman method also for veterinary diagnostics and highlights the broader potential implication for zoonotic pathogen monitoring and for elucidating the molecular and cellular basis of biological processes in a non-invasive manner.

We showed that Raman spectroscopy can differentiate developmental states of *C*. *abortus*, both as isolated forms and within intact host cells. This differentiation is based on differences in their biochemical compositions. Raman spectroscopic imaging uses these differences to generate high-quality false color Raman images with diffraction-limited spatial resolution of intact infected host cells that provide label-free subcellular insight into the niche of the intracellular pathogen. The EB and RB morphoforms of *C*. *abortus* can be visualized, localized and quantified directly within their host cells. Due to the spatial resolution limit, individual EBs were not resolved, only aggregates/groups of several cells and more. Both methods, TEM and Raman imaging, can provide quantitative insights into the status of infection and provide valuable information on the infection cycle.

## Experimental procedures

### 2D cell culture infection model

BGM cells from the Collection of Cell Lines, obtained from the Friedrich-Loeffler-Institut - Federal Research Institute for Animal Health Germany, site Riems, were used as host cells for *Chlamydia* infection. *Chlamydia abortus* S26/3 was obtained from the *Chlamydia* strain collection of the German National Reference Laboratory of Enzootic Abortion of Ewes at the Friedrich-Loeffler-Institut, site Jena. The cells were regularly tested for *Mycoplasma* using PromoKine PCR *Mycoplasma* Test Kit II (Promocell).

### Preparation of isolated *Chlamydia abortus* morphoforms

BGM cells were seeded in 75 cm^2^ cell culture flasks and incubated in Eagle's Minimum Essential Medium (EMEM, Lonza) at 37 °C and 5% CO_2_ for 3 days. Cells were infected with *Chlamydia abortus* S26/3 at a multiplicity of infection (MOI) of 10 in 14 ml UMDCK medium (Lonza) per flask. The cells were then incubated at 37 °C and 5% CO_2_ for 36 h for the isolation of RBs, and for 54 h for the isolation of EBs, harvested in 1.5 ml SPGA medium and stored at −80 °C. After thawing, infected cells were sonicated twice for 30 s at 4 °C. Cell debris was removed by centrifugation at 1000 × *g*/10 min/4 °C and the supernatant was centrifuged at 25,800 × *g*/40 min/4 °C to collect the chlamydial pellet. After resuspension of the pellet in 1 ml PBS, sonication and differential centrifugation steps at 1000 × *g* and 25,800 × *g* were repeated and the final pellet was fixed in 500 μl 4% methanol-free formaldehyde (Image-iT, Invitrogen) overnight at 4 °C. The pellet was collected by centrifugation at 25,800 × *g*/40 min/4 °C and washed twice in 1.5 ml sterile Purelab water. After a last centrifugation step at 25,800 × *g*/40 min/4 °C, the pellet was re-suspended in 20 μl sterile Purelab water. The same isolation procedure was performed with uninfected BGM cells. The fragments from this isolation were analyzed as control samples. The isolation procedure was performed with two independent biological samples, in the following called batches.

### Characterization of isolated *Chlamydiaabortus* morphoforms

For negative contrast TEM preparation, suspensions were vortexed for 5 min and droplets of 30 μl were placed on a plate of dental wax. 300-mesh copper grids, that had been filmed with formvar, coated with carbon and hydrophilized by glow discharge, were floated on the droplets for 10 min. Then, grids were briefly rinsed with distilled water and contrasted on a drop of 1% uranyl acetate for 1 min. Grids were examined by transmission electron microscopy (Tecnai 12, FEI Deutschland GmbH) at 80 KV. Representative micrographs were taken on sheet film, developed and digitized using a transmitted light scanner. The size of the chlamydiae was measured using the EM-Measure software (TVIPS).

An aliquot of the isolated EBs was subjected to scanning electron microscopy. For this, small droplets of suspensions were placed on glass coverslips. After 30 min of sedimentation, the coverslips with attached EBs were fixed with freshly prepared modified Karnovsky fixative (4% w/v paraformaldehyde, 2.5% v/v glutaraldehyde in 0.1 M sodium cacodylate buffer, pH 7.4) for 1 h at room temperature. After washing 3 times for 15 min each with 0.1 M sodium cacodylate buffer (pH 7.4), the samples were dehydrated in ascending ethanol concentrations (30, 50, 70, 90 and 100%) for 15 min each. Next, the samples were critical-point dried using liquid CO_2_ and sputter coated with gold (thickness approx. 2 nm) using a CCU-010 sputter coater (safematic GmbH). Finally, the specimens were investigated with a field emission SEM LEO-1530 Gemini (Carl Zeiss NTS GmbH).

For Raman analysis, a droplet of 10 μl of the respective suspension was placed in the middle of a CaF_2_-slide (Crystal GmbH) and allowed to dry at room temperature. Image scans with step size of 0.087 μm (most with 6 × 6 μm^2^ with 69 *x* 69 points^2^, some with 5 × 5 μm^2^ with 57 *×* 57 points^2^ or 7 × 7 μm^2^ with 80 *x* 80 points^2^) of the dried isolated *Chlamydia* morphoforms (and the fragments from uninfected BGM cells) were recorded with 1 s integration time per pixel at different sample positions using an upright confocal Raman microscope alpha300 R (WITec GmbH). The Raman device was equipped with a frequency-doubled Nd:YAG-laser (532 nm) yielding 15 μm in the sample plane. The Raman signal was collected through a 100 x objective (NA 0.75, Carl Zeiss Microscopy GmbH) and guided through a 100 μm fiber.

### Sample preparation and transmission electron microscopy for intracellular life cycle analysis

BGM cells in T75 flasks were infected with *Chlamydia abortus* S26/3 at an MOI of 10 in 14 ml UMDCK medium per flask, incubated at 37 °C and 5% CO_2_. At 18, 36, 48 and 54 h post infection (h p.i.), cells were fixed in 2.5% glutaraldehyde in cacodylate buffer (0.1 M, pH 7.2) with 1.8% glucose for 2 h at 4 °C. Then, they were gently scraped off the culture plate, transferred to an Eppendorf tube and the fixative replaced by cacodylate buffer. For embedding, cells were centrifuged for 5 min at 1,500 *x g*. The cell pellet was embedded in 2% agarose and sectioned to 1 mm^3^ cubes. Cubes were post-fixed in 2% osmium tetroxide, dehydrated in acetone and embedded in araldite Cy212. Relevant areas were selected in Toluidine blue-stained semi-thin sections. Ultrathin sections (80 nm) were stained with uranyl acetate and lead citrate, examined by transmission electron microscopy (Tecnai 12; FEI), and representative micrographs documented (TEMCAM FX416, TVIPS, Gauting, Germany).

*Chlamydia abortus* morphoforms in each TEM image generated were counted according to the rules specified in Supplemental Table S1. At least ten different individual inclusions were analyzed per time point, in particular 20 inclusions for 18 hpi, 10 inclusions for 36 hpi and 48 hpi each, and 11 inclusions for 54 hpi.

### Sample preparation for intracellular life cycle analysis by Raman spectroscopy

CaF_2_ microscope slides were incubated overnight in 70% ethanol, rinsed with EMEM and placed in 24-well cell culture plates. BGM cells were seeded on the slides in a concentration of 5 x 10^4^ cells per slide and incubated in EMEM at 37 °C and 5% CO_2_ for 3 days. They were infected with *Chlamydia abortus* S26/3 in UMDCK medium at an MOI of 50 and incubated for 18, 36, 48 and 56 h. Medium was removed and *Chlamydia*-infected cells were washed with PBS before they were fixed with 4% methanol-free formaldehyde (Image-iT, Invitrogen, Thermo Fisher Scientific) for 20 min at room temperature. After removal of the fixative, the samples were washed with PBS and stored in 1 ml PBS at 4 °C.

For faster detection of infected cells, the inclusions were stained with antibodies. First, the samples were washed three times with PBS and then incubated for 10 min in permeabilization buffer and then for 1 h in block buffer. The cells were then incubated for 1 h with the primary antibody (Anti-*Chlamydia trachomatis* + *psittaci* Antibody LS- C128098 from LSBio) at a concentration of 5.46 μg/ml. After washing five times with permeabilization buffer, incubation with the secondary antibody (DyLight 405, IgG mouse, dianova) took place for 1 h. The cells were washed five times with permeabilization buffer and then stored lightproof in 1 ml PBS at 4 °C.

### Raman spectroscopic measurements of intracellular *Chlamydiaabortus*

Raman measurements were performed with 532 nm excitation (15 mW in the sample plane) on an upright confocal Raman microscope alpha300 R used in an earlier study (20). Intact, infected BGM cells were analyzed directly in aqueous medium (PBS) with a 60× water immersion objective (NA 1, Nikon). Individual cells with inclusions were selected from the bright field image. In order to achieve highest spatial resolution in z, a 25 μm fiber served as pinhole. Raman maps were recorded with a step size of 69 nm covering an area of 10 × 10 μm^2^ with 145 pixels × 145 pixels. Three layers were recorded with a spacing of 1 μm in z (one layer above the focal plane and one below), yielding an imaged region of 10 × 10 × 3 μm^3^. Integration time per pixel was 1 s.

### Analysis of Raman data

Pre-processing and analysis of Raman data was done by R (34) using packages “hyperSpec” (https://github.com/r-hyperspec/hyperSpec) (35), “MASS” (36), “Spikes” (37), and “unmixR” (https://github.com/r-hyperspec/unmixR/releases/tag/v2.6). Packages “ggplot2” (38), “cowplot” (39), and “ggh4x” (https://github.com/teunbrand/ggh4x) (40) were used for visualization. Prior to analysis, Raman spectra were preprocessed by removing cosmic spikes, cutting out the silent region between 1800 cm^-1^ and 2700 cm ^-1^, correcting baseline, and excluding unspecific or outlier spectra.

Raman images of both, isolated and intracellular *C*. *abortus*, were analyzed using linear hyperspectral unmixing. The main idea of the approach is to decompose the original spectral data into pure components (so-called endmembers) and corresponding abundances. For this, first, we applied principal component analysis (PCA) for dimension reduction. Then, we extracted endmember (EM) spectra from the images using N-FINDR algorithm (41). Finally, using the EM spectra, abundances were calculated using non-negative least squares followed by sum normalization.

Number of endmembers was chosen using an approach applied in a previous work (42): an excessive number of endmembers was used in N-FINDR; then the EMs and their abundances were grouped based on assignment. The endmember spectra were assigned to specific components considering the characteristic Raman bands and abundance distribution in the image. False color abundance maps were produced by assigning a color to each endmember and mixing them according to abundances.

### Raman analysis of isolated morphoforms

Raman data of isolated morphoforms were additionally processed by normalization and resampling the wavenumbers using Locally Estimated Scatterplot Smoothing (43) for interpolation. First, we applied PCA and N-FINDR to Raman images of *C*. *abortus* isolated by the default protocol only (18 h, 24 h, 36 h, and 48 h post infection) in order to extract endmembers. Ten endmember spectra were extracted by N-FINDR and then grouped. Redundant endmembers were replaced by a single endmember calculated as median. The EMs were used to calculate abundances for the same images and, for comparison, isolated *C*. *abortus* with protocol optimized to isolate EBs (labeled as EB), and fragments from uninfected BGM cells (labeled as BGM).

### Multivariate data analysis of Raman maps of infected BGM cells

After excluding measurements with low signal from cells and/or bacteria (*i*.*e*., spectra dominated by PBS/water from the surrounding medium), 8 Raman images from three z-stacks were retained for analysis (20, 41). We first applied PCA and N-FINDR to extract 16 endmembers. These endmembers were then assigned to the following components: EB, RB, host-cell DNA (*e*.*g*., nucleus), host-cell cytoplasm, and PBS/water from the surrounding medium. Remaining endmembers represented irrelevant artefacts (*e*.*g*., residual cosmic ray spikes, autofluorescence, plastics) and were not considered in the analysis. Abundance maps were computed in two steps. First, abundances were estimated using non-negative least squares for each EM. Second, abundances of endmembers belonging to the same component were summed to yield the total abundance per component. These were the values used for the false color images and analysis of component distribution.

## Data availability

Data are available upon request from the corresponding author.

## Supporting information

This article contains supporting information.

## Conflict of interest

The authors declare that they have no conflicts of interest with the contents of this article.
