# Supplementary material for: Insights into chlamydial infection at the sub-cellular level using label-free Raman spectroscopy in comparison to electron microscopy
Source: J Biol Chem. 2025 Nov 13;302(1):110940. doi: 10.1016/j.jbc.2025.110940 (PMC12743436; doi:10.1016/j.jbc.2025.110940)
Supplement: Supporting Material [file mmc1.pdf]

Supplementary information

Insights into chlamydial infection at the sub-cellular level using label-free Raman spectroscopy in comparison to electron microscopy

Nancy Unger [a,b], Elisabeth M. Liebler-Tenorio [c], Rustam R. Guliev [b], Simone Eiserloh [a,b,d], Sandor Nietzsche [d,e], Frauke Nowak [c], Sara Zuchantke [c], Christian Berens [c], Christiane Schnee [c], Ute Neugebauer [a,b,d,f] *

[a] *Center for Sepsis Control and Care, Jena University Hospital, 07747 Jena, Germany*
[b] *Leibniz Institute of Photonic Technology, Member of Leibniz Health Technologies, Member of the Leibniz Centre for Photonics in Infection Research (LPI), 07745 Jena, Germany*
[c] *Friedrich-Loeffler-Institut – Federal Research Institute for Animal Health (FLI), Institute of Molecular Pathogenesis, 07743 Jena, Germany*
[d] *ThIMEDOP – CeTraMed, Jena University Hospital,07747 Jena, Germany*
[e] *Center for Electron Microscopy, Jena University Hospital, 07743 Jena, Germany*
[f] *Friedrich Schiller University Jena, Institute of Physical Chemistry and Abbe Center of Photonics, 07743 Jena, Germany*

Content

Supplementary Figure S1: N-FINDR analysis of the Raman image scans of isolated *C. abortus* morphoforms 2
Supplementary Figure S2: Electron microscopy images showing infected BGM cells at different time points post infection 6
Supplementary Figure S3: False colour images of 3D Raman image stacks of infected BGM cells at time points 18 h, 36 h, 48 h and 54 h post infection (p.i.). 7
Supplementary Figure S4: Abundance distribution of RBs and EBs in Raman image stacks 7
Supplementary Figure S5: Masks applied to 3D Raman image stacks of infected BGM cells to calculate abundance distributions. 8
Supplementary Figure S6: Histogram representation of abundance distributions of RBs and EBs from Raman data. 9
Supplementary Table S1: Rules for counting different *C. abortus* morphoforms in TEM images 10
Supplementary Table S2: Results of quantitative image analysis of the TEM images 11
Supplementary Table S3: Comparison of conventional transmission electron microscopy (TEM) and Raman spectroscopy 12

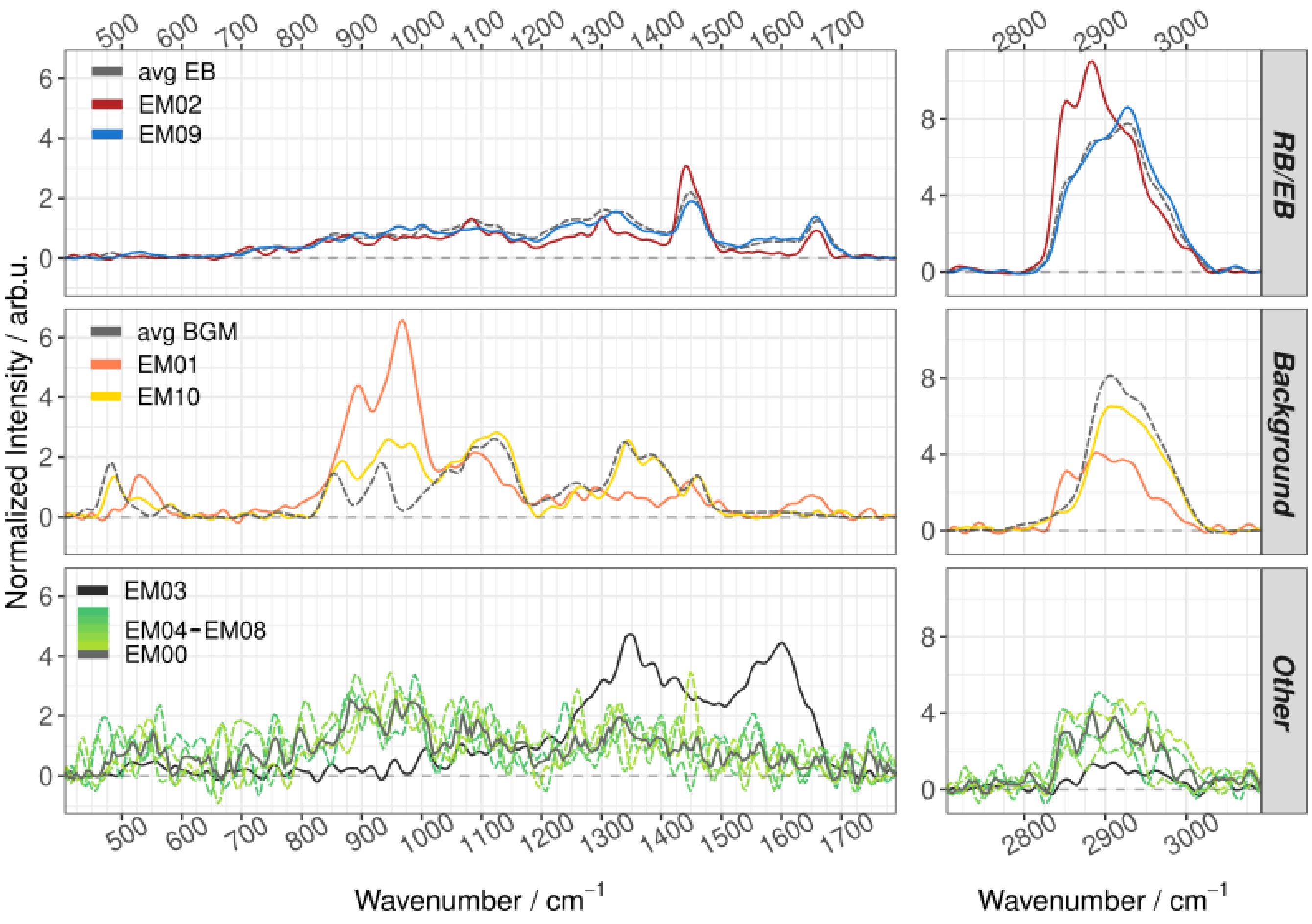

Supplementary Figure S1-A: N-FINDR analysis of the Raman image scans of isolated *C. abortus* morphoforms.

Endmember spectra revealed from N-FINDR analysis of the Raman image scans of *C. abortus* isolates at different time points post infection. Dashed lines indicate additional spectra that were not used for abundance calculation.

1st row: EM 02 (red) and EM 09 (blue) show clear spectral features of bacteria and were assigned to RB (red) and EB (blue), as discussed in the main manuscript. For comparison, the dashed grey line pictures the mean spectrum of EB morphoform from the optimized EB isolation protocol.

2nd row: EM 01 (orange) and EM 10 (yellow) show Raman spectra of background components that were introduced during isolation. E.g. EM 01 shows typical components of carbohydrates that are present during density gradient centrifugation. For comparison, the dashed grey line pictures the mean spectrum of the “isolates” obtained when performing the isolation procedure on uninfected BGM cells (avg. BGM).

3rd row: EM 04 – EM 08 (dashed shades of green) reveal low intensity spectra that were grouped together into single endmember EM 00 (gray) by taking the median. EM 03 (black) shows unspecific spectra that were present at only very few pixels and have not been considered any further.

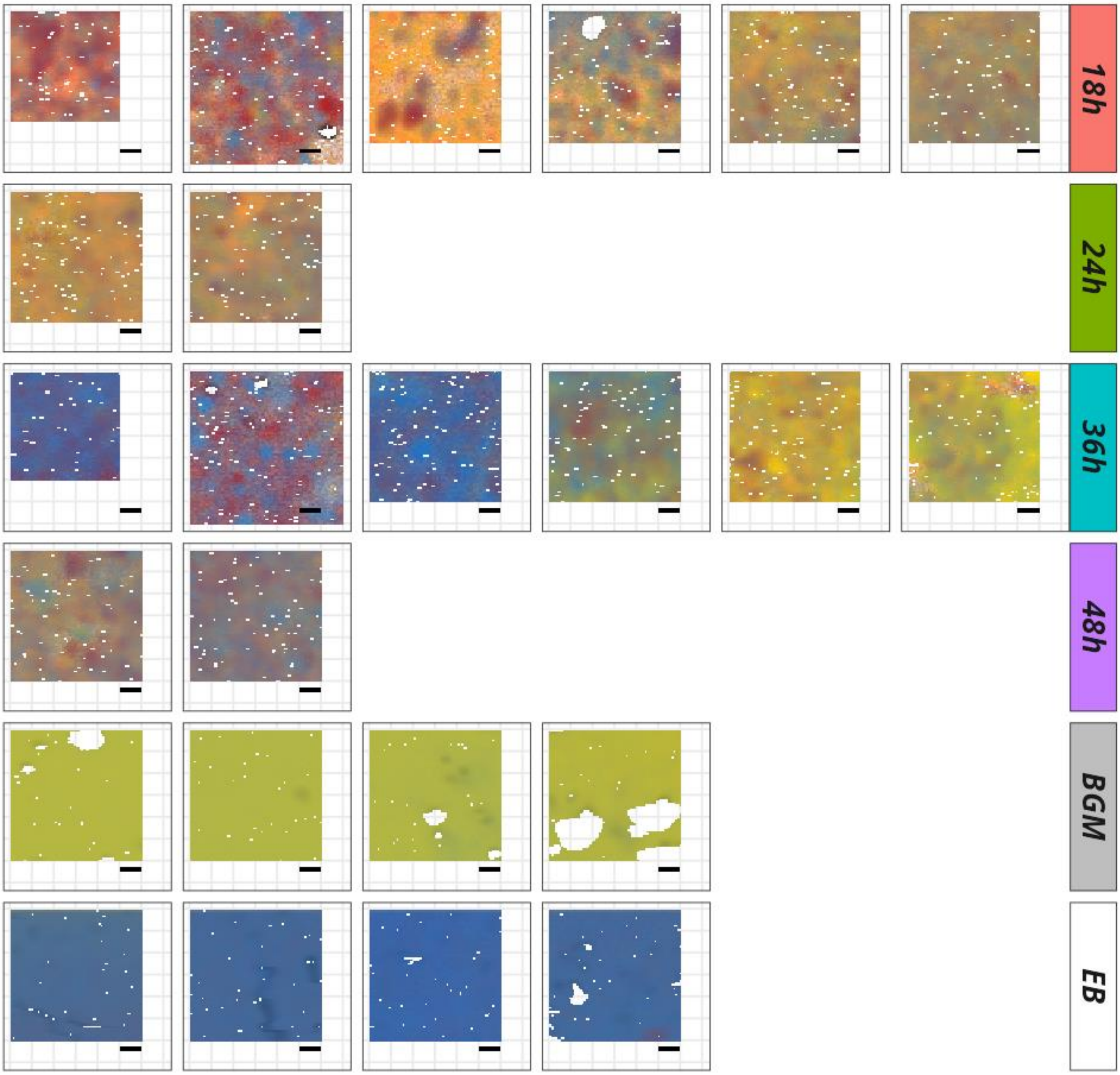

Supplementary Figure S1-B: N-FINDR analysis of the Raman image scans of isolated *C. abortus* morphoforms.

Abundance maps for EM shown with solid lines in Supplementary Figure S1-A. All endmembers were assigned the same colours as in Supplementary Figure S1-A, except for EM 00 which was now assigned white color for the false colour images. Abundances were calculated using Non-Negative Least Squares (NNLS) with normalization. Images (row name – column number): 18h-2, 36h-2, 48-2, EB-2 are also shown in Figure 1 E- 1H in the main manuscript. Scale bars are 1µm. Each outer panel square corresponds to an area of 7 µm x 7 µm.

Panel S2A: 18 hpi

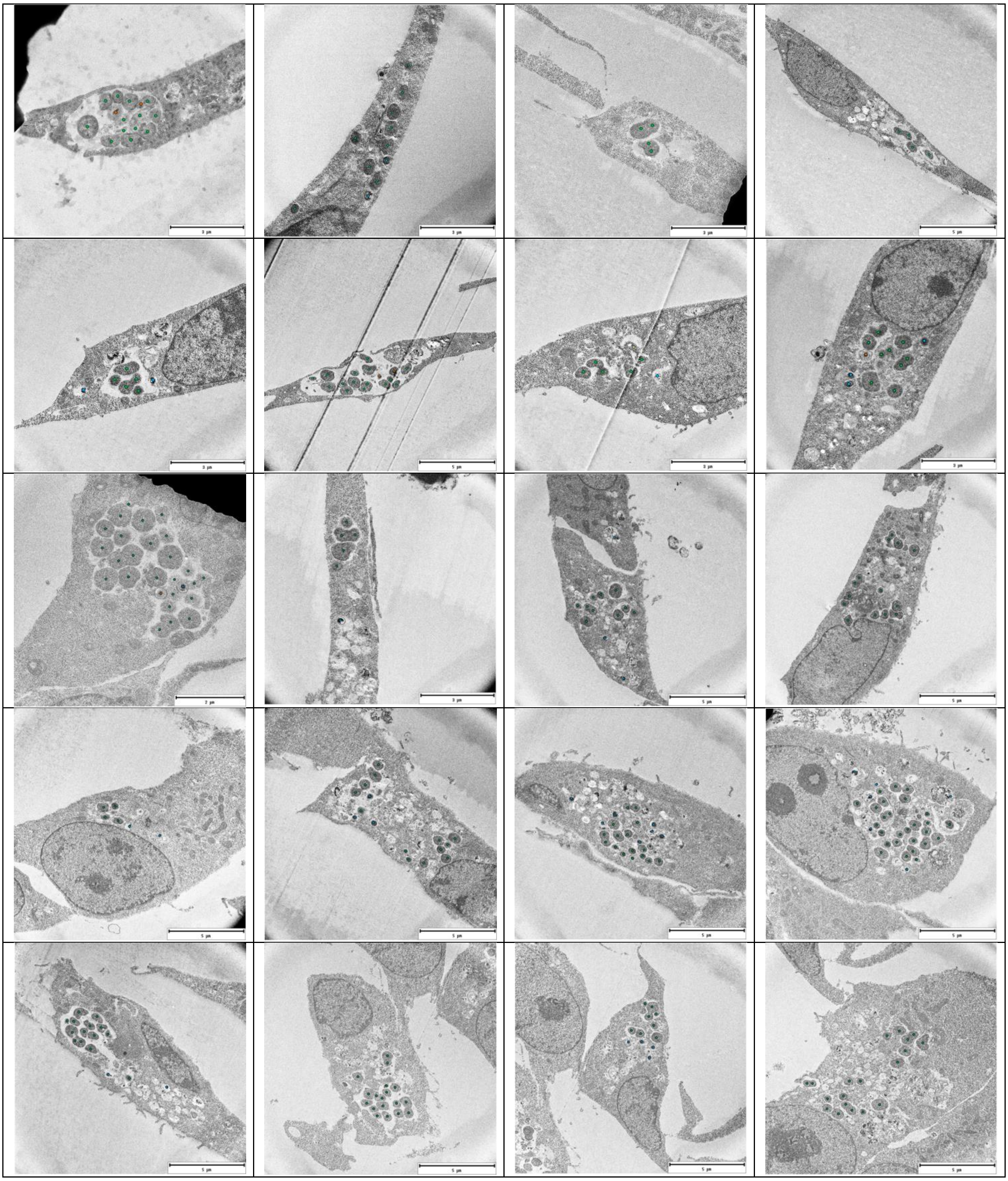

Panel S2B: 36 h p.i.

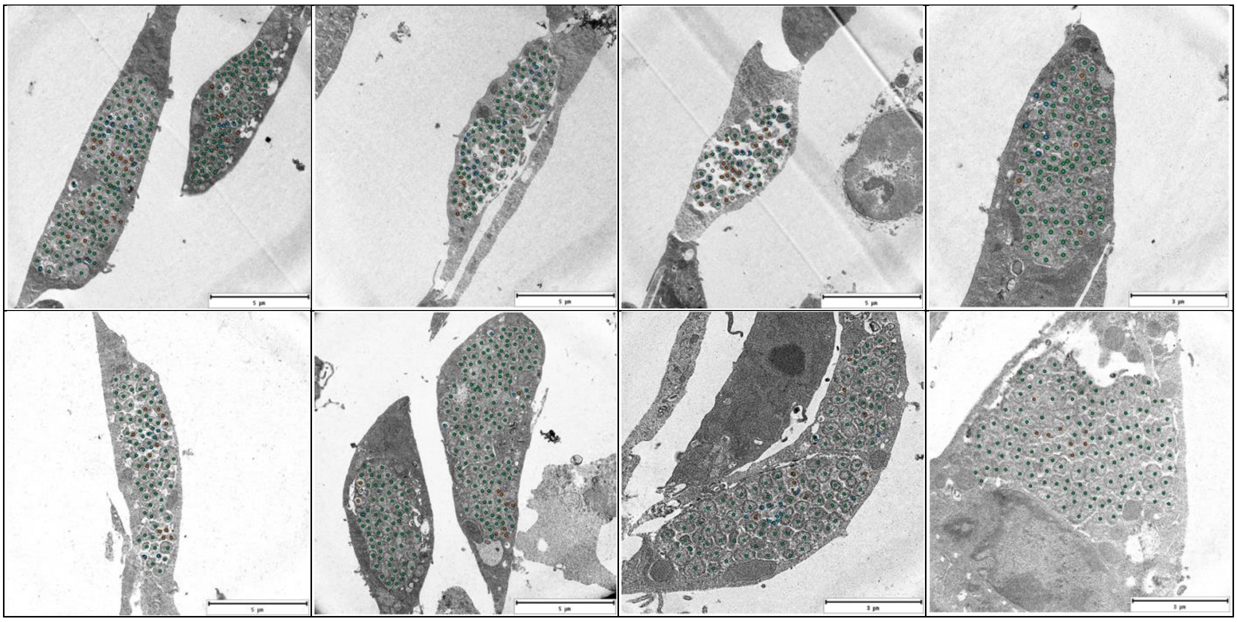

Panel S2C: 48 h p.i.

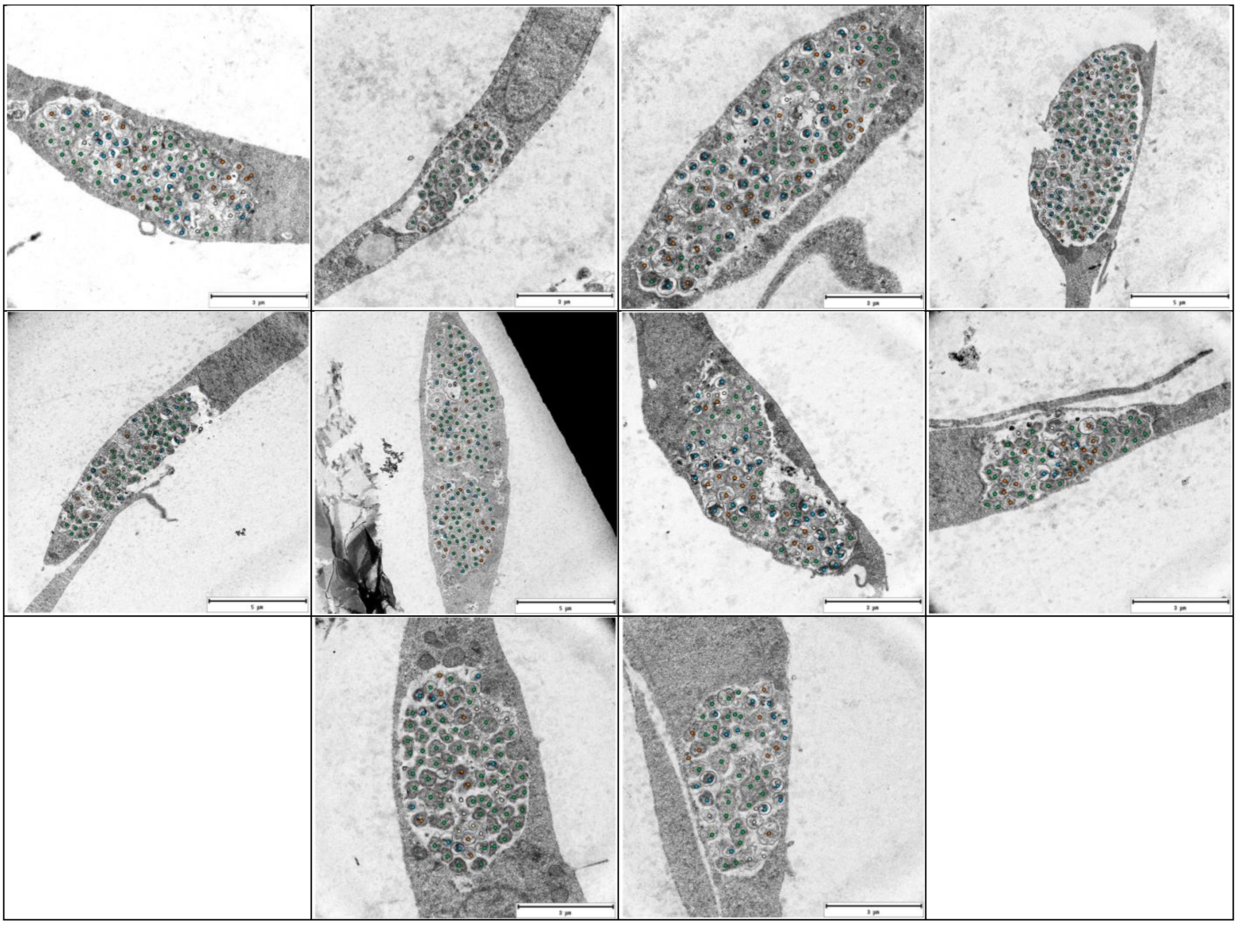

Panel S2D: 54 h p.i.

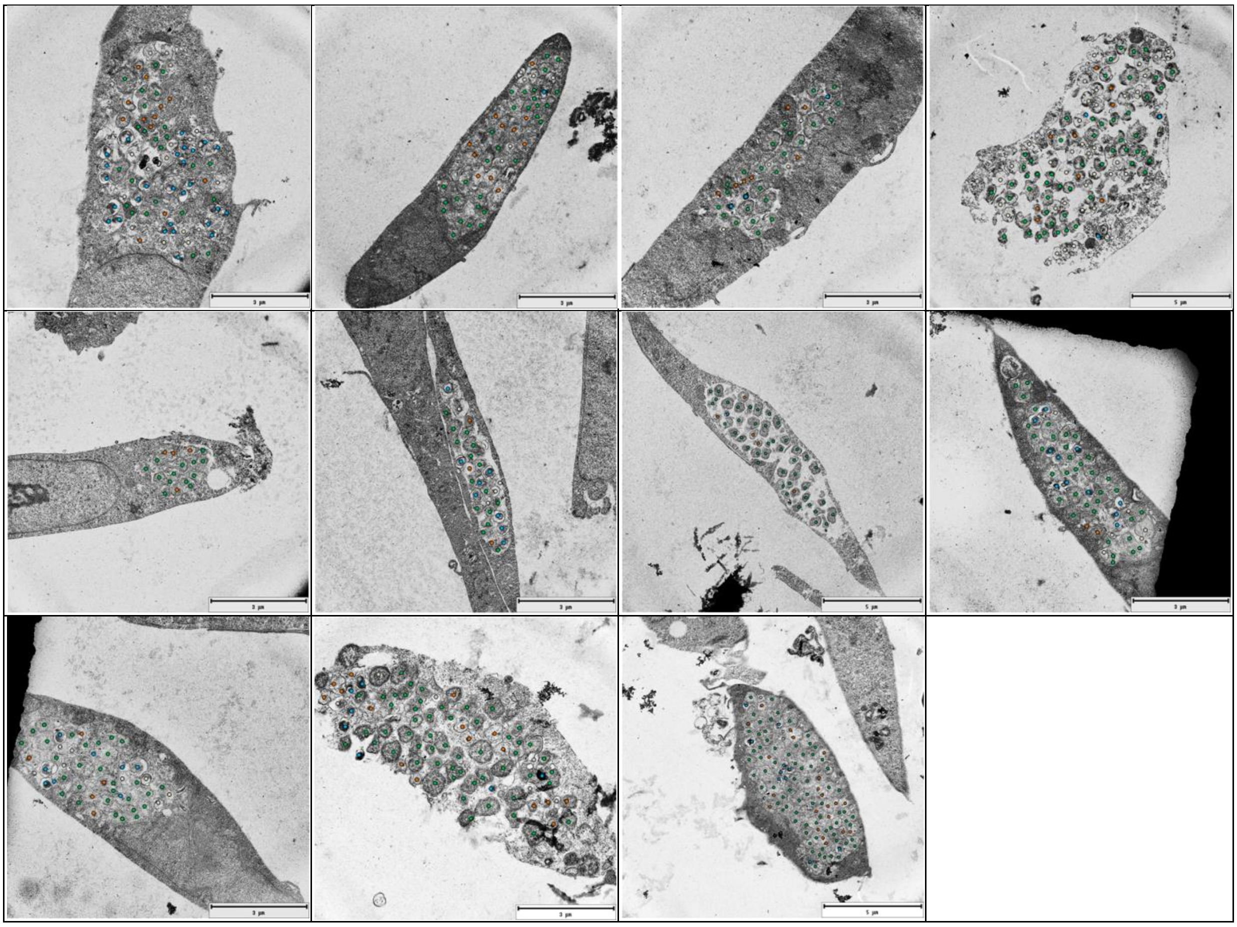

Supplementary Figure S2: Electron microscopy images showing infected BGM cells at different time points post infection (p.i.) with C. abortus. **S2A**: 18 hours p.i.; **S2B**: 36 hours p.i.; **S2C**: 48 hours p.i.; **S2D**: 54 hours p.i. Coloured dots indicate assignment: green labels RBs, blue labels EBs, red labels IBs and white labels undefined morphoforms/material in inclusions. Criteria to assign RBs, EBs and IBs are summarized in Supplementary Table S1 and the counted values are displayed in Supplementary Table S2. This figure shows all images analyzed in this study, including the EM images displayed in Figure 2, main manuscript.

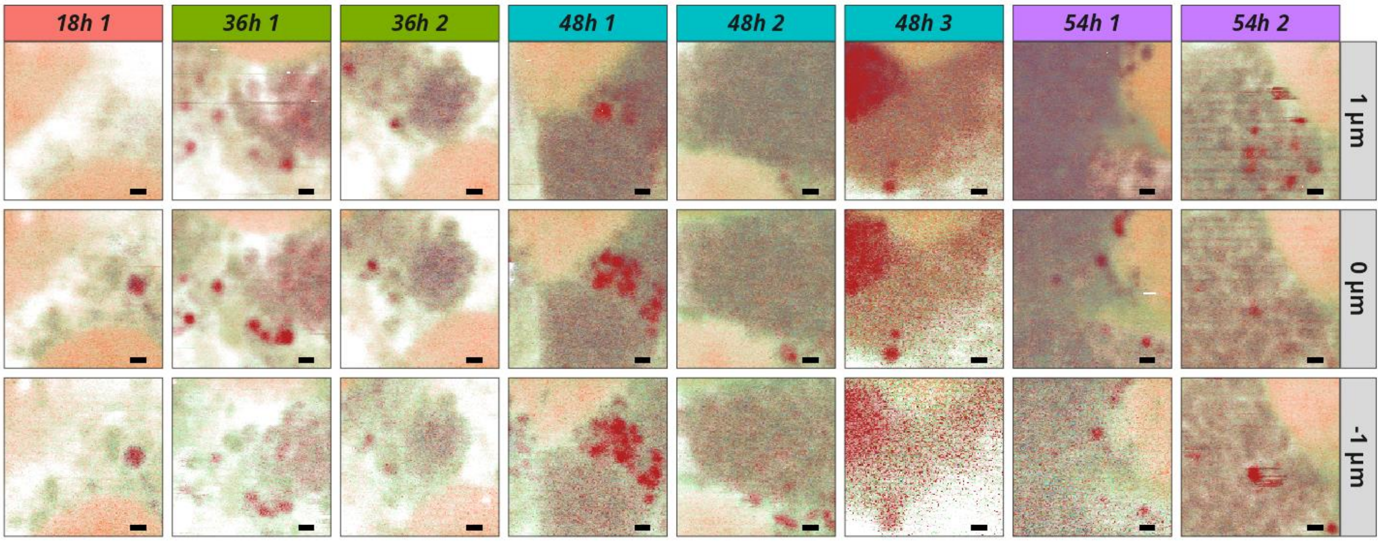

Supplementary Figure S3: False colour images of 3D Raman image stacks of infected BGM cells at time points 18 h, 36 h, 48 h and 54 h post infection (p.i.). False colour images of all eight three z-planes Raman images. Each corresponds to a specific measurement labelled by hours post infection (h p.i.) and an index number indicating different cells; each row corresponds to a z-layer. The colour code and endmembers are same as in Figure 3, main manuscript: white - PBS/water, orange - DNA/nucleus, dark red - RB of *C. abortus*, mid blue - EB of *C. abortus*, light green - cytoplasm of the host cell. Each false colour image is a superposition of the relative abundance of each endmember in each pixel. Scale bars are 1 µm. Measurements “18h 1”, “36h 1”, “48h 2”, and “54h 1” are shown in Figure 3B, main manuscript as a z-stack projection.

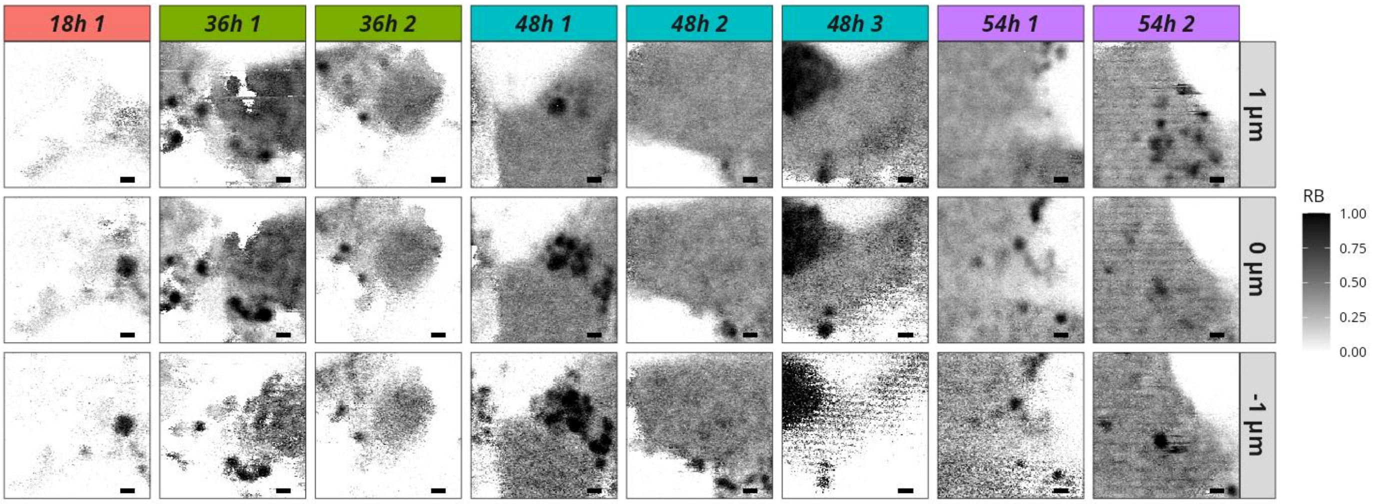

Supplementary Figure S4: Abundance distribution of RBs and EBs in Raman image stacks

Supplementary Figure S4-A: Abundances distribution of RB component in the image scans shown in Supplementary Figure S3 as grey scale images. The panels 0 µm of “18h 1”, “36h 1”, “48h 2”, and “54h 1” are also shown in Figure 3C, main manuscript.

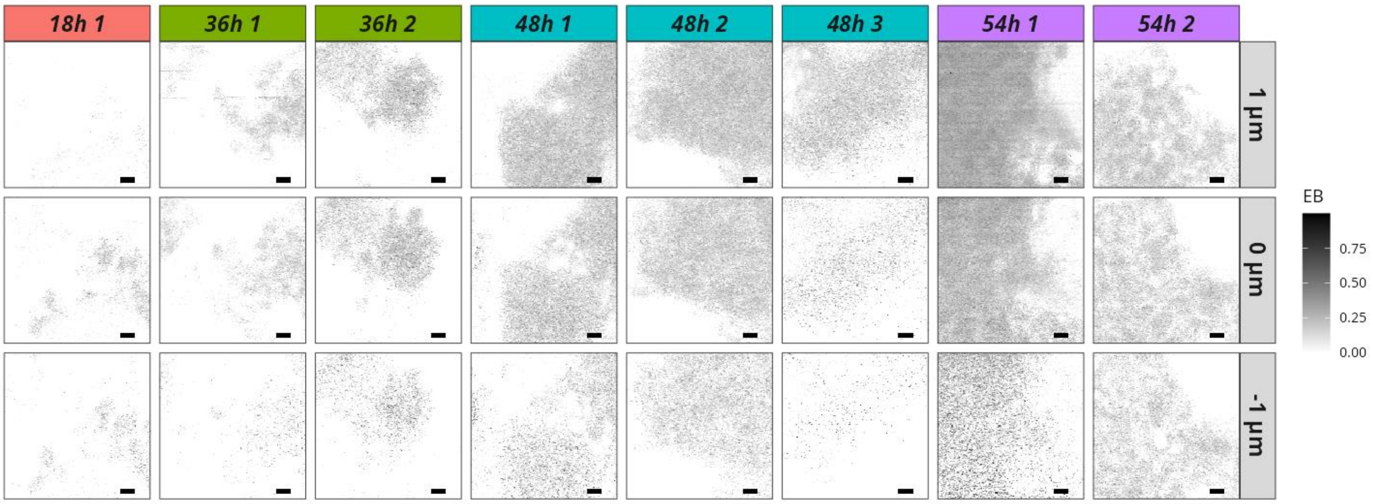

Supplementary Figure S4-B: Abundances distribution of EB component in the image scans shown in Supplementary Figure S3 as grey scale images. The panels 0 µm of "18h 1", "36h 1", "48h 2", and "54h 1" are also shown in Figure 3C, main manuscript.

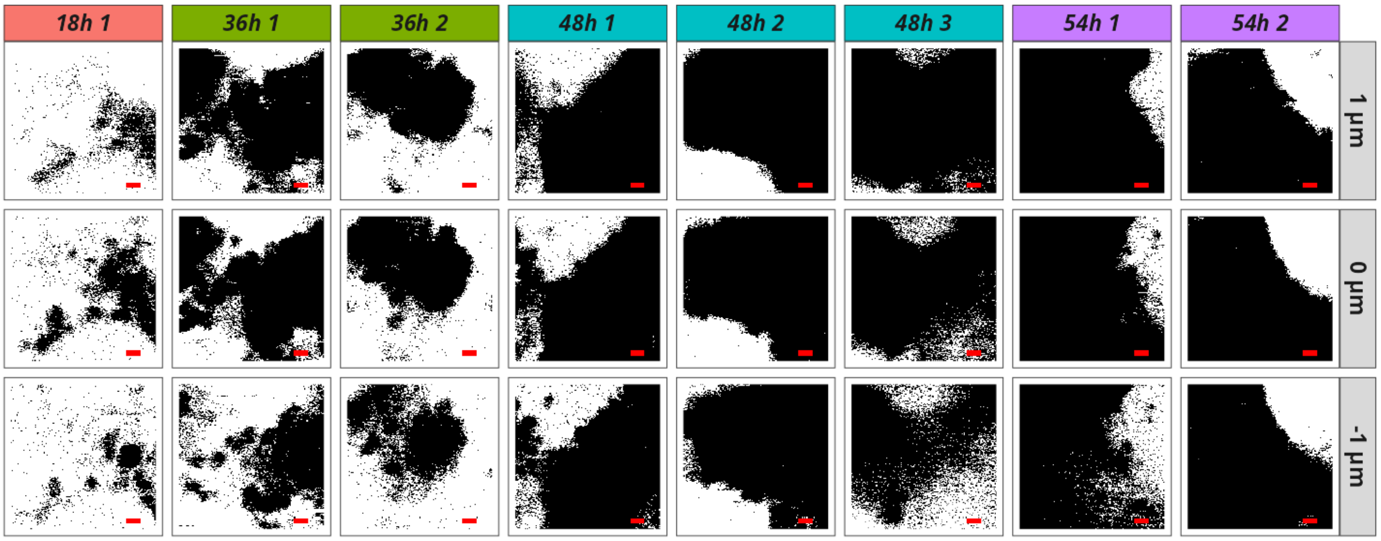

Supplementary Figure S5: Masks applied to 3D Raman image stacks of infected BGM cells to calculate abundance distributions. Masks applied to Raman images in Supplementary Figure S3 to calculate abundance distributions shown in Figure 4, main manuscript. Black pixels indicate the regions that we used for calculation. The applied threshold is that total abundance of both EB and RB must be ≥ 5%. White regions were excluded. This originates mainly from host cell nuclei (orange regions in Supplementary Figures S3, or background (white regions in Supplementary Figure S3).

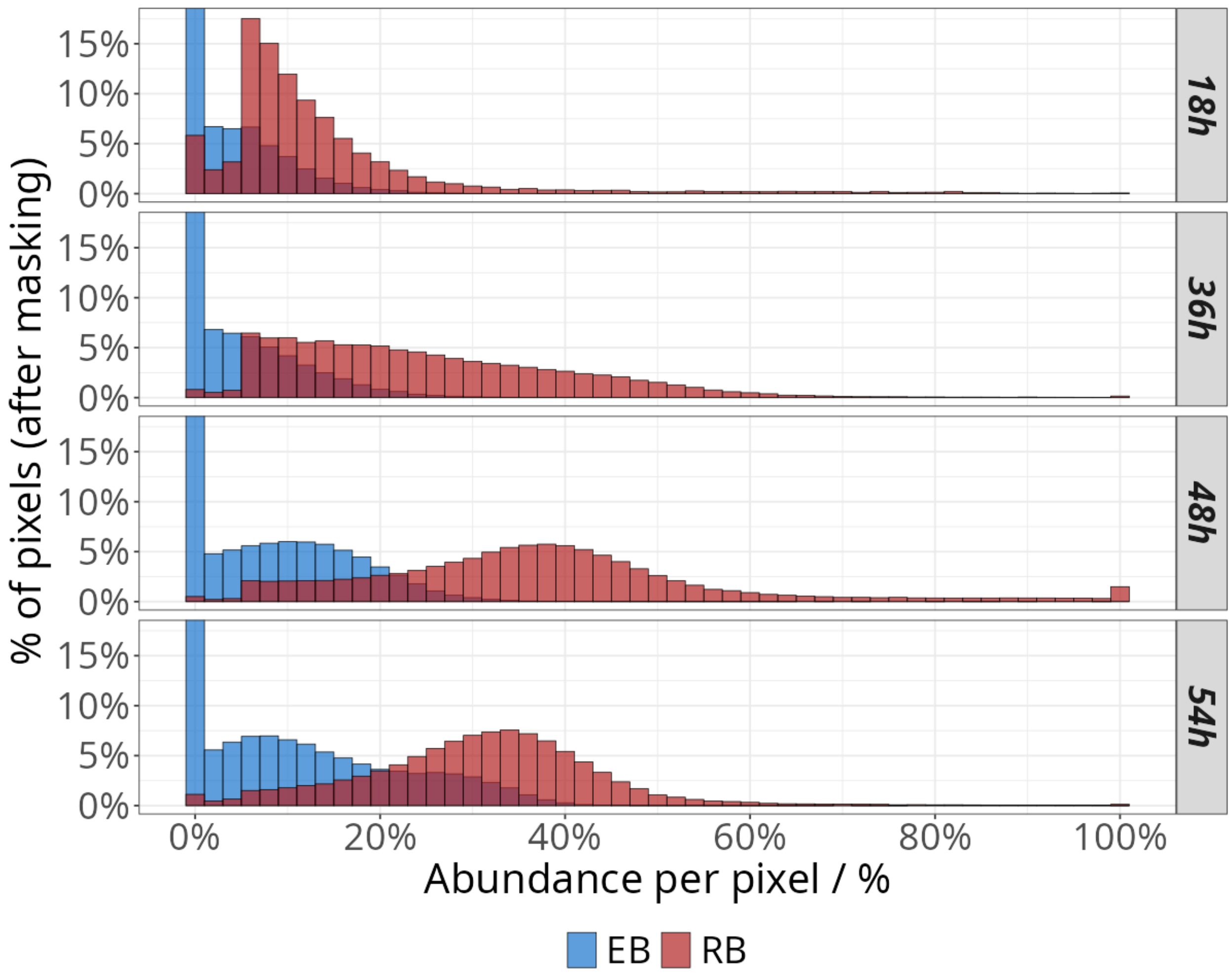

Supplementary Figure S6: Histogram representation of abundance distributions of RBs and EBs from Raman data. The histogram representation shows the same data as depicted in Figure 4B, main manuscript, in a different form of visualization. The distribution of RB (red) and EB (blue) endmember abundances is presented, combining data from all 8 Raman image 3 D stacks. Abundance values are scaled from 0 - 1 to 0 - 100 to be represented in percentages. The images were masked so that only pixels with less than 5% of total RB and EB contribution were excluded, i.e. excluding irrelevant PBS/water and nucleus regions. Applied masks are shown in Supplementary Figure S4. Due to the large number of pixels with 0% EB abundance, the y axis is cut to 17% for better visibility. The values for the clipped blue (EB) bar at 0%: 18h - 65%, 36h - 60%, 48h - 41%, 54h - 21%.

Supplementary Table S1: Rules for counting different *C. abortus* morphoforms in TEM images**.**

C. abortus morphoform	Morphological features / criteria
Elementary body (EB)	- round shape<br>- diameter of 0.2 to 0.3 µm<br>- electron-dense<br>- often distinct periplasmic space
Reticular bodies (RB)	- round to oval to pleomorphic shape<br>- size between 0.3 and 1.14 µm<br>- less electron-dense than EBs, homogeneous, finely granular cytoplasm<br>- RBs in replication count as two RBs if distinct constriction or separated by membrane
Intermediate body (IB)	- round shape<br>- size between EBs and RBs<br>- electron-lucent with small (approx. 0.1 µm) electron-dense core
Not clearly categorizable bacteria (NCCB)/material	- chlamydia that cannot clearly be categorized as a morphoform<br>- membrane-bound material of variable shape and variable interior, e.g. multiple electron-dense granules

Supplementary Table S2: Results of quantitative image analysis of the TEM images to reveal the number of the different *C. abortus* morphoforms at different hours post infection (h p.i.). The criteria for counting EBs, IBs, RBs and NCCB listed Supplementary Table S1 were used.

Time point (hpi)	Cell	Number of inclusions	Absolut amount of EBs[1]	Absolut amount of IBs[1]	Absolut amount of RBs[1]	Absolut amount of NCCB[1]	Relative amount of RB[2] (%)[1]	Relative amount of EB[2] (%)
18 hpi	1	1	0	1	13	0	92.9	0
	2	5	2	0	9	0	81.8	18.2
	3	2	0	0	3	0	100.0	0
	4	3	0	0	4	0	100.0	0
	5	5	3	0	5	0	62.5	37.5
	6	1	0	1	17	0	94.4	0
	7	3	0	1	6	1	85.7	0
	8	8	2	1	10	0	76.9	15.4
	9	1	0	2	25	0	92.6	0
	10	2	1	0	4	0	80.0	20.0
	11	9	7	0	10	0	58.8	41.2
	12	7	0	0	14	0	100.0	0
	13	4	3	0	4	0	57.1	42.9
	14	8	3	3	13	0	68.4	15.8
	15	10	6	1	17	0	70.8	25.0
	16	9	3	3	27	0	81.8	9.1
	17	4	3	0	15	0	83.3	16.7
	18	3	0	0	18	0	100.0	0
	19	6	2	0	6	0	75.0	25.0
	20	9	3	2	19	0	79.2	12.5
						**average ± std**	**82.1 ± 13.8**	**14 ± 14.6**
36 hpi	1	3	15	14	120	5	80.5	10.1
	2	1	7	0	87	1	92.6	7.4
	3	1	13	2	91	5	85.8	12.3
	4	1	14	0	41	4	74.5	25.5
	5	2	6	0	97	0	94.2	5.8
	6	1	7	2	75	0	89.3	8.3
	7	1	1	2	65	3	95.6	1.5
	8	2	1	2	145	0	98.0	0.7
	9	2	7	4	105	0	90.5	6.0
	10	1	0	4	135	0	97.1	0
						**average ± std**	**89.8 ± 7.6**	**7.8 ± 7.4**
48 hpi	1	1	28	1	56	7	32.9	65.9
	2	1	2	2	29	1	6.1	87.9
	3	1	36	5	61	8	35.3	59.8
	4	1	40	0	89	16	31.0	69.0
	5	1	11	0	65	3	14.5	85.5
	6	2	26	4	106	7	19.1	77.9
	7	1	31	4	33	9	45.6	48.5
	8	1	5	4	42	3	9.8	82.4
	9	1	17	4	63	14	20.2	75.0
	10	1	15	4	25	8	34.1	56.8
						**average ± std**	**70.9 ± 13.1**	**24.9 ± 12.8**
54 hpi	1	1	28	6	25	16	42.4	47.5
	2	1	1	0	47	1	97.9	2.1
	3	1	3	1	22	3	84.6	11.5
	4	1	3	9	54	35	81.8	4.5
	5	1	0	3	13	0	81.3	0
	6	1	13	0	15	4	53.6	46.4
	7	2	1	3	43	0	91.5	2.1
	8	2	12	1	31	6	70.5	27.3
	9	1	11	1	31	8	72.1	25.6
	10	1	5	13	61	6	77.2	6.3
	11	1	12	9	68	3	76.4	13.5
						**average ± std**	**75.4 ± 12.2**	**17 ± 17.4**

1: chlamydial particles were counted per inclusion (see also Supplementary Figure S2 for respective electron microscopy images

Supplementary Table S3: Comparison of conventional transmission electron microscopy (TEM) and Raman spectroscopy for the analysis of intracellular bacteria. Both methods are able to generate insightful images that yield information on the composition of the chlamydial inclusions. (a condensed form of the table is also presented in the main manuscript, Table 2).

**Parameter**	**Conventional TEM***	**Raman spectroscopic imaging**
**Principle of the method**	Electrons pass through ultrathin sample sections; interaction with atoms results in scattering of the electron beam	Inelastic light scattering on molecular vibrations
**Imaging contrast due to**	High atomic numbers of the contrasting agents (U, Os, Pb)	Different chemical compositions with different molecules yielding different vibrations
**Condition of sample**	Chemically fixed samples embedded in resin (60 nm sections)	Here: fixed intact cell, but live-cell imaging is also possible
**Sample preparation**	Duration: <2 weeks involving several preparation steps (fixation, dehydration, resin-embedding, ultrathin sectioning, contrasting)	Minimal sample preparation is required.<br>Grow cells on CaF_2 slides to achieve minimal spectral background from substrate,<br>Chemical fixation with formalin/paraformaldehyde can preserve the cells for longer periods of time, but live-cell imaging is also possible
**Speed of data acquisition**	Microscopy, acquisition of micrographs of selected areas <1 s	~ 1s/pixel (2 h per image: 6 h per stack) (technological improvements are expected from true imaging set-ups that are currently under development)
**Spatial resolution**	< 0.1 nm, atomic resolution achieved, despite the fact that the theoretical resolution limit is not reached in conventional TEM due to aberration of the lenses. The theoretical limit depends on the de Broglie wavelength of the electrons:<br>$d = \lambda_e/2n \sin\alpha$<br>with $\lambda_e = h/\sqrt{2mE\left(1+\frac{E}{2mc^2}\right)}$<br>where h is Planck´s constant, m is the rest mass of an electron, E is kinetic energy of the accelerated electron	Follows the Abbe limit:<br>$d = \lambda/2NA$. With excitation at 532 nm, and NA=1, d > 266 nm.<br>3D imaging with resolution in z-direction of ~500 nm is possible as whole cell is still present
**Data analysis**	Visual identification, image analysis: numbers of RBs, EBs, IBs/region of interest (cell, chlamydial inclusion)	Several multivariate statistical algorithms exist to extract the relevant information from the spectral data. Here, NFINDR and PCA-LDA were used.
**Information content**	Morphology of host cell (membranes, cytoplasm, organelles, nucleus); morphology of chlamydial inclusions (size, shape, localisation in relation to	Biochemical composition (without the need of any labels/stains),<br>Size and shape within Abbe's resolution limit.

	organelles/membranes of host cell); number, size, morphology of morphoforms (RBs, EBs, IBs)	False colour images<br>Differentiation between different morphoforms possible, including quantitative analysis

* more advanced methods, such as HRTEM, STEM, Cryo Substitution TEM, TEM tomography, and Atomic Force Microscopy are not considered.
